# From photoprotection to plasticity: transposon activation in the *Chlamydomonas det1* mutant

**DOI:** 10.1111/nph.70436

**Published:** 2025-08-07

**Authors:** Konomi Fujimura‐Kamada, Jun Minagawa

**Affiliations:** ^1^ Department of Environmental Photobiology National Institute for Basic Biology 38 Nishigonaka, Myodaiji Okazaki 444‐8585 Japan; ^2^ Basic Biology Course Graduate Institute for Advanced Studies, SOKENDAI 38 Nishigonaka, Myodaiji Okazaki 444‐8585 Japan

**Keywords:** green algae, NPQ, photosynthesis, stress response, transposable element, ubiquitin E3 ligase

## Abstract

- Transposable elements (TEs) contribute to genomic adaptation but are typically silenced to maintain genome integrity. In this study, we investigated TE activation in a *Chlamydomonas reinhardtii* mutant deficient in DE‐ETIOLATED1 (DET1). This mutant was originally identified for its enhanced high‐light tolerance due to constitutive nonphotochemical quenching (NPQ). While NPQ mitigates light‐induced stress, its persistent activation compromises growth under low light (LL).
- Notably, the slow‐growing *det1* cultures under LL conditions rapidly reverted to a fast‐growing phenotype. The recurrent emergence of fast‐growing suppressor mutants indicated a previously unrecognized role of DET1 in TE suppression. To explore this possibility, we performed phenotypic, molecular, and genomic analyses, including TE insertion mapping and gene expression studies in *det1* and its suppressor mutants.
- Our analysis uncovered that the phenotypic suppression resulted from the insertion of a specific TE, *Bill*, into the subunits for a transcription factor for the photoprotective genes *LHCSR1*/*3*, CrCO/NF‐Ys. These insertions disrupted NPQ, restored efficient light harvesting, and facilitated growth in LL.
- These results suggest that DET1 integrates NPQ induction for photoprotection and TE mobilization for genomic plasticity, bridging short‐term responses with long‐term adaptation.

## Introduction

There are two types of transposable elements (TEs) classified based on their mechanism of transposition: retrotransposons (Class I), which use an RNA intermediate and perform copy‐and‐paste transposition, and DNA transposons (Class II), which do not involve an RNA intermediate and perform cut‐and‐paste transposition (Feschotte & Pritham, 2007). DNA transposons are the first TE discovered with the genetic characterization of the Ac/Ds family of autonomous and nonautonomous TEs in maize (McClintock, 1950). Over billions of years of evolution, TEs have struck a balance between detrimental effects on individuals and long‐term beneficial effects on species through increasing genomic variations (Kazazian Jr., 2004). The activation of TE occurs under specific conditions, such as stress, encompassing environmental, biological, and chemical factors (McClintock, 1984). Numerous studies have enhanced our understanding of how stress escalates transposon activity, primarily through alterations in epigenetic markers and modifications in chromatin structure (Slotkin & Martienssen, 2007; Zeh *et al*., 2009; Casacuberta & Gonzalez, 2013). Such an increase in transposon activity under stress conditions may contribute to an increase in genetic diversity within a population, potentially bolstering the species' adaptive capacity in response to new environmental challenges (McClintock, 1984; Feschotte & Pritham, 2007; Casacuberta & Gonzalez, 2013; Raingeval *et al*., 2024). Despite these insights, the underlying molecular mechanisms by which environmental stress leads to elevation of transposon activity remain largely elusive.

In this study, we investigated a mutant of the green unicellular alga *Chlamydomonas reinhardtii* lacking DE‐ETIOLATED 1 (*det1*). This mutant was previously isolated for its unique high‐light (HL)‐tolerant characteristics (Aihara *et al*., 2019). Photosynthetic organisms are in general susceptible to HL stress despite their ability to harness light energy because it promotes the production of reactive oxygen species. Their defense mechanism against such stress involves activating nonphotochemical quenching (NPQ), a process that thermally dissipates excess light energy (Minagawa, 2013). In *C. reinhardtii*, the expression of components necessary for NPQ is generally low under low‐light (LL) conditions. When exposed to light stress, such as HL/UV, the suppression of these components is relieved, leading to the induction of NPQ. Central to this process is the induction and activation of photoprotective proteins including LIGHT‐HARVESTING COMPLEX STRESS RELATED1 (LHCSR1) and LHCSR3 (Allorent & Petroutsos, 2017). Expression of these LHCSRs is triggered by blue light perception through the phototropin pathway (Petroutsos *et al*., 2016) and UV light perception through the UVR8 pathway (Allorent *et al*., 2016), respectively. Because the phototropin mutant, *phot*, is unable to induce LHCSR3, it is unable to grow under HL conditions (Petroutsos *et al*., 2016). The *phot det1* mutant was identified as a suppressor of *phot* (Aihara *et al*., 2019). It displayed enhanced expression of LHCSRs, resulting in efficient growth under HL (NPQ > 4.0).

The transcription of *LHCSRs* is positively regulated by a transcription factor complex comprising CONSTANS (CO) and the NUCLEAR FACTOR‐Y (NF‐Y) B and C subunits, whose activity is inhibited by the ubiquitin E3 ligase complex CONSTITUTIVE PHOTOMORPHOGENIC1 (COP1)/SUPPRESSOR OF PHYA‐105 1 (SPA1) (Gabilly *et al*., 2019; Tokutsu *et al*., 2019a). The activity of COP1/SPA1 is, in turn, downregulated upon exposure to HL or UV (Gabilly *et al*., 2019; Tokutsu *et al*., 2019a). Additionally, DET1, a component of another ubiquitin E3 ligase, CULLIN 4 (CUL4)–UV‐DAMAGED DNA BINDING PROTEIN 1 (DDB1)^DET1^, has been suggested to enhance COP1/SPA1 (Lau & Deng, 2012; Aihara *et al*., 2019). Thus, when DET1 is inhibited upon exposure to HL, the transcription of *LHCSRs* is induced. A current model of the signal transduction pathways involved in NPQ induction in *C. reinhardtii* is presented in Supporting Information Fig. S1.

In the literature, DET1 was initially reported as a negative regulator of photomorphogenic development in *Arabidopsis thaliana*, in which the *det1* mutant manifests a de‐etiolated phenotype in darkness (Pepper *et al*., 1994). The function of DET1 in land plants has been described as enhancing the activity of COP1/SPA1 ubiquitin E3 ligase (Lau & Deng, 2012), a role similarly observed in *C. reinhardtii*. Furthermore, DET1 is a highly conserved protein present in not only plants but also mammals. For example, in human embryonic kidney cells, DET1 promotes the ubiquitination and degradation of the proto‐oncogenic transcription factor c‐Jun (Wertz *et al*., 2004), suggesting that it is involved in diverse cellular processes, including oncogenic transformation (Shaulian & Karin, 2002; Wakabayashi *et al*., 2011).

## Materials and Methods

### Algal strains and growth conditions

*Chlamydomonas reinhardtii* Dangeard strain CC‐125 (137c mt+) was obtained from the Chlamydomonas Resource Center (https://www.chlamycollection.org/) and was used as wild‐type (WT) strain. The *phot det1* mutant, derived from a cell‐wall‐less Dangeard strain (cw15), was isolated in a previous study (Aihara *et al*., 2019). The *phot* single mutant based on CC‐125 (Greiner *et al*., 2017) was generously provided by Prof. Peter Hegemann (Humboldt University of Berlin). The previously obtained *CrCO*, *NFYB*, and *NFYC* mutants (*crco‐2*, *nfyb‐1*, and *nfyc*) (Tokutsu *et al*., 2019b) were crossed with the *det1* CRISPR‐Cas9 mutant (will be discussed later) to generate double mutants: *det1 crco‐2*, *det1 nfyb‐1*, and *det1 nfyc*, respectively. Cells were cultured mixotrophically at 25°C under 40–50 μmol photons m^−2^ s^−1^ in tris‐acetate‐phosphate (TAP) medium (Gorman & Levine, 1965) unless otherwise stated. For NPQ measurements and immunoblot analysis of LHCSR proteins, cells were precultured in TAP medium under 40–50 μmol photons m^−2^ s^−1^ until mid‐logarithmic phase (1–2 × 10^6^ cells ml^−1^). Cells were then resuspended in Sueoka's high salt medium (Sueoka, 1960). After an incubation under LL conditions (10–15 μmol photons m^−2^ s^−1^) for *c*. 24 h with shaking, the cell concentration was adjusted to an OD_680_ at between 0.40 and 0.44. Subsequently, cells were exposed to either LL or HL (*c*. 400 μmol photons m^−2^ s^−1^) for 4 h. White fluorescent lamps were used as the LL source, while an LED panel, KR93SP (380–700 nm; Eco‐lamps Inc., Hong Kong, China), was used as the white HL source.

### Generation of the *det1* mutant via CRISPR‐Cas9

CRISPR‐Cas9 system was employed to target exon 5 of the *DET1* gene to create *det1* mutants using a guide RNA (5′—GGATGTCGTACAGTTCATGG—3′) following a previously established protocol (Tokutsu *et al*., 2019a). A double‐stranded tag‐V2 donor (ds‐tag‐V2) was used in place of a homology‐directed repair donor. Recombinant *Streptococcus pyrogenes* Cas9 (spCas9) protein, tracrRNA, and crRNA were purchased from Integrated DNA Technologies (Coralville, IA, USA). Cells of the *phot* mutant based on CC‐125 grown under synchronized light cycles were transformed with a ribonucleoprotein complex (spCas9, tracrRNA, and crRNA), ds‐tag‐V2, and PCR‐amplified *AphVIII* cassette (conferring paromomycin‐resistant), using the NEPA21 Super Electroporator (Nepa Gene Co., Ltd, Ichikawa, Japan). Transformed cells were selected on TAP plates containing 10 μg ml^−1^ paromomycin under dim light at 25°C. Paromomycin‐resistant colonies were screened for the insertion of the ‘FLAG’ sequence, situated in the middle of the tag‐V2, using whole‐genome PCR with primers DET1‐check‐F, DET1‐check‐R, FLAG ver2‐check‐F, and FLAG ver2‐check‐R. The sequences of tag‐V2 and PCR primers are listed in Table S1. The genomic region surrounding the CRISPR target site in the positive clone (E8) was PCR‐amplified using primers DET1‐check‐F and DET1‐check‐R and subjected to sequencing analysis to characterize the mutant structure. The *det1* mutant was established after four rounds of backcrossing with the WT.

### Growth phenotype analysis

Cells were suspended in TAP medium at 5 × 10^6^ cells ml^−1^. Five microliters of droplets of fivefold serial dilutions of cell cultures was spotted onto TAP plates and incubated at 25°C.

### Isolation of *dos* suppressor mutants

The *phot det1* mutant was crossed with the WT. Among the 19 progeny retaining the *det1* genotype, four exhibiting a faster growth rate under the light intensity at 40–50 μmol photons m^−2^ s^−1^ were isolated (7A: mt−, 8B: mt−, 7C: mt+, and 3H: mt+). Two independent single colonies from each isolated progeny were tested for NPQ to assess whether the faster growth rate under the light intensity at 40–50 μmol photons m^−2^ s^−1^ correlated with reduced NPQ relative to the WT. These suppressor mutations were named *dos1* in 7A, *dos2* in 8B, *dos3* in 7C, and *dos4* in 3H (*det* one suppressor). Among newly derived *det1* progeny generated by crossing the *det1* single mutant with other related mutants, one line exhibiting a significantly faster growth rate than the original *det1* single mutant was designated as *dos5*.

### Immunoblot analysis

SDS‐PAGE and immunoblot analysis were performed as previously described (Takahashi *et al*., 2006). Briefly, total cellular proteins were solubilized in 2% SDS and 0.1 M dithiothreitol at 100°C for 3 min, separated by SDS‐PAGE using a resolving gel containing 6 M urea, and transferred onto a PVDF, or polyvinylidene fluoride, membrane. The membrane was then probed with an anti‐LHCSR antibody (Tokutsu *et al*., 2019b) and visualized using enhanced chemiluminescence.

### Genetic linkage analysis of the mutants

The *det1 dos* mutants were crossed with each other. To assess whether the resultant progeny have a ‘*dos* phenotype’, they were subjected to NPQ measurement. Progeny that did not show high NPQ under LL conditions were considered to have a ‘*dos* phenotype’. Genetic linkage analysis revealed the existence of two linkage groups among the five *dos* mutations: one comprising *dos1*, *dos2*, and *dos3*, and another comprising *dos4* and *dos5*. Similarly, genetic crosses between *det1 dos1* and *det1 nfyc*, *det1 dos1* and *det1 crco*, and *det1 dos5* and *det1 nfyb* were conducted, and the resultant progeny were subjected to NPQ measurement. These results are summarized in Table S2.

### Chl fluorescence quenching analysis

Chl fluorescence was measured essentially as previously described (Tokutsu *et al*., 2019a) using a FluorCam 800MF imaging fluorometer (Photon Systems Instruments, Drásov, Czech Republic). Cells were transferred to a 48‐well culture plate and adapted under far‐red light (< 5 μmol photons m^−2^ s^−1^) for 15 min before measurements. Actinic light irradiation was applied at 750 μmol photons m^−2^ s^−1^. NPQ values were calculated as NPQ = (F_m_ – F_m_′)/F_m_′.

### Southern blot analysis

Genomic DNA (3 μg) was digested using restriction enzymes, subjected to electrophoresis on a 0.7% agarose gel, and transferred to a positively charged nylon membrane (cat no. 11209299001, Roche Diagnostics, Basel, Switzerland). A digoxigenin (DIG)‐labeled probe for detecting the *Bill* sequence was prepared as follows: A *c*. 1.3 kb DNA fragment containing the *Bill* insert was PCR‐amplified using primers CON1‐F2 and CON1‐R2, with whole‐genome DNA of the *det1 dos3* mutant as the template. This PCR product was used as a template for a second round of PCR using primers *Bam*HI‐Bill‐Fw and *Eco*RI‐Bill‐Rv. The resulting 576‐bp DNA fragment was digested with *Bam*HI and *Eco*RI and cloned into the pBluescript II KS+ vector (Agilent Technologies, Inc., Santa Clara, CA, USA), generating pBSIIKS‐Bill plasmid. Following sequence confirmation of the *Bill* insert, the pBSIIKS‐Bill plasmid was used as a template to produce the DIG‐labeled probe. The 576‐bp fragment containing the *Bill* sequence was PCR‐amplified with primers BamHI‐Bill‐Fw and EcoRI‐Bill‐Rv using a random primer labeling kit (DIG‐High Prime DNA Labeling and Detection Starter Kit II, Roche). Electrophoresis, blotting, hybridization, and signal detection were performed according to the manufacturer's instructions.

### Whole‐cell genomic PCR for genotyping

Genomic DNA was extracted as follows. Forty microliters of cell cultures grown in TAP medium to a late‐logarithmic phase, or a similar number of cells grown on a TAP plate, was collected by centrifugation and resuspended in 25 μl of a TE10:10 solution (10 mM Tris–HCl, 10 mM sodium EDTA, pH 8.0). The cell suspension was heated to 99°C for 8 min, immediately transferred to ice, and diluted with 100 μl of sterile water. This cell suspension was frozen in a freezer at −30°C or in liquid N_2_. The frozen sample was thawed and used as a PCR template. PCR was performed using KOD FX Neo polymerase (TOYOBO Co., Ltd, Osaka, Japan). The PCR primer sequences are provided in Table S1.

### Examination of *Bill* insertion into the *NFYB* and *CrCO* genes

Cells were suspended in TAP medium at *c*. 2 × 10^6^ cells ml^−1^ to establish the ‘Start culture’. The start culture was diluted 1 : 10 000 in two flasks, each containing 100 ml of TAP medium. Cells were cultured with shaking under 40–50 μmol photons m^−2^ s^−1^ light. Cultures that reached a concentration of 2–7 × 10^6^ cells ml^−1^ were referred to as the first passage. The first passage was diluted 1 : 10 000 in the same manner, and the culture that again reached 2–7 × 10^6^ cells ml^−1^ were referred to the second passage. The second passage was diluted 1 : 10 000 in the same manner, and the resultant culture that reached 2–7 × 10^6^ cells ml^−1^ was referred to as the third passage. For WT strain, the third passage was further diluted 1 : 10 000 and the resultant culture that reached 2–7 × 10^6^ cells ml^−1^ was referred to as the fourth passage. From each passage culture, 2 × 10^5^ cells were collected for PCR tests in the same manner as described in ‘Whole‐cell genomic PCR for genotyping’ in the Materials and Methods section.

## Results

### *det1* mutant exhibits growth defect under LL

**Fig. 1:**
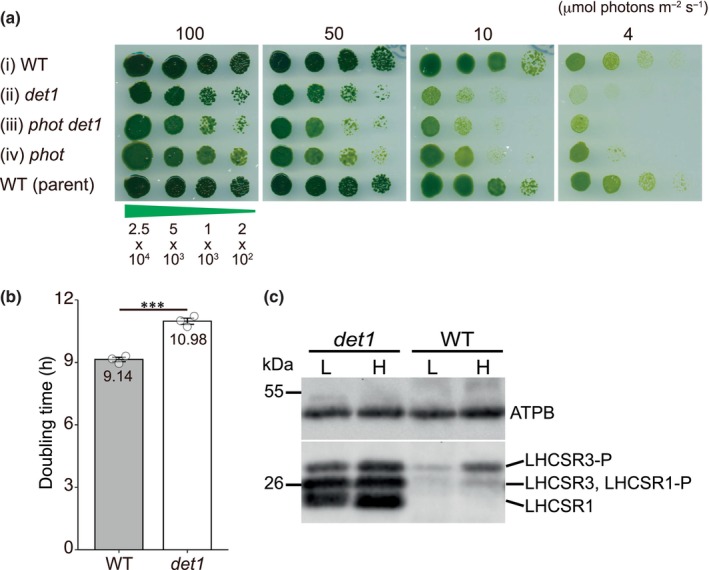
Slow growth of the *Chlamydomonas reinhardtii* DE‐ETIOLATED 1 (*det1*) mutant under low‐light (LL) conditions. (a) Growth comparison of *det1* mutants (*phot det1* and *det1*) with the wild‐type (WT). A complete progeny set from a single tetrad of the fourth backcross ((i)–(iv)), along with the parental WT, was incubated for 7 d under various light intensities, as indicated above each panel. The numbers below the panels represent the cell counts spotted on tris‐acetate‐phosphate (TAP) plates. (b) Doubling time of the *det1* mutant under a light intensity of 50 μmol photons m^−2^ s^−1^. Cells were grown in TAP medium with shaking. Data represent the mean ± SEM (*n* = 3). Statistical significance was assessed using Welch's *t*‐test (***, *P* < 0.001). (c) Immunoblot analysis of LIGHT‐HARVESTING COMPLEX STRESS RELATED (LHCSR) proteins in the *det1* mutant. Whole‐cell extracts containing 1 μg of Chl were prepared from cells exposed to LL (L; 10 μmol photons m^−2^ s^−1^) or high light (H; 400 μmol photons m^−2^ s^−1^) for 4 h, and loaded into each SDS‐PAGE lane. AtpB (beta subunit of ATP synthase) levels serve as a loading control.

In *C. reinhardtii*, although the suppressor mutant *phot det1* exhibits remarkable tolerance to HL, its growth is compromised in LL due to the reduced photosynthetic quantum yield caused by its high NPQ capacity (Wilson *et al*., 2023). Intriguingly, the slow‐growing *phot det1* cultures consistently transition into fast‐growing cultures after repeated subculturing in LL, propagating at rates similar to the WT. This implies that the *det1* population employs an adaptation strategy to ensure the species survival, leveraging the inherent stress responses to HL. To investigate the molecular mechanism underlying this adaptation strategy, we generated a *det1* single mutant by crossing the previously isolated *phot det1* double mutant with WT. The initial approach was hindered by the cell‐wall‐less (*cw15*) characteristics of the original double mutant. As an alternative strategy, we employed the CRISPR‐Cas9 system to introduce a *det1* insertion mutation into the cell‐walled *phot* mutant (Greiner *et al*., 2017). The resulting *phot det1* double mutant was backcrossed with the WT four times (Fig. S2B). The single *det1* mutant derived from the final backcross was used for the subsequent studies. The *det1* single mutant exhibited slower growth rates under light intensities lower than 50 μmol photon m^−2^ s^−1^ (Fig. 1a), with *c*. 20% longer doubling time when compared to the WT (Fig. 1b). This mirrored the characteristics of the original *phot det1* double mutant (Wilson *et al*., 2023). LHCSRs were expressed higher than the WT under both HL and LL conditions, supporting the high NPQ phenotype in the *det1* mutant (Fig. 1c).

### Emergence of *dos* mutants as *det1* suppressors

**Fig. 2:**
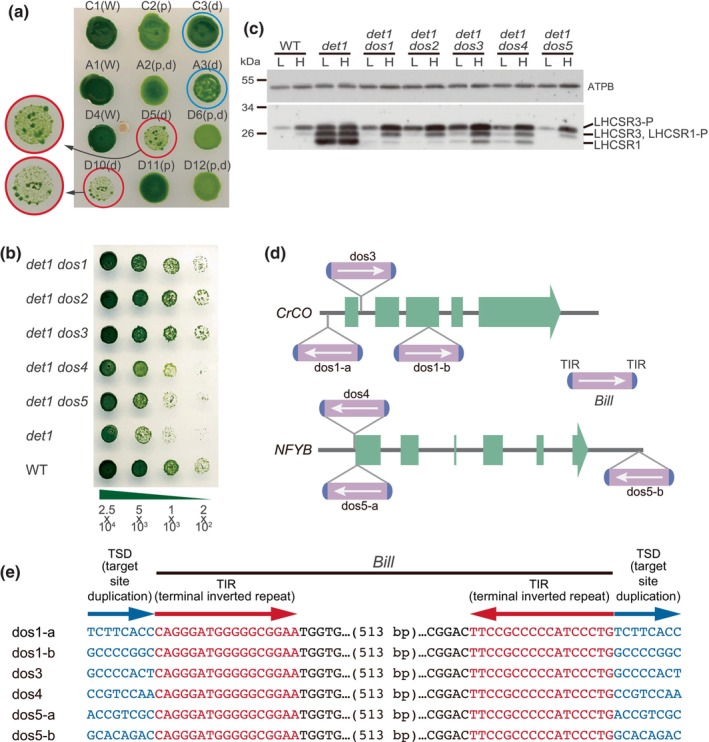
Characterization of the *det1* suppressor (*dos*) mutants in *Chlamydomonas reinhardtii*. (a) Growth of the DE‐ETIOLATED 1 (*det1*) F3 progenies. Five microliters of cell suspensions at 1 × 10^6^ cells ml^−1^ was spotted on a tris‐acetate‐phosphate (TAP) plate and incubated for 17 d under 4 μmol photons m^−2^ s^−1^. The names of the clones are shown above their spots with genotype in parentheses. ‘p’, ‘d’, and ‘W’ denote *phot*, *det1*, and wild‐type (WT), respectively. Clones with relatively good growth (C3, A3) are marked with *cyan circles*, while poorly grown *det1* clones (D5, D10) are marked with *red circles*. Enlarged photographs of the red‐marked clones show several larger colonies. (b) Growth comparison of the *det1 dos* mutants, *det1* mutant, and WT. Cells were incubated for 4 d under 50 μmol photons m^−2^ s^−1^. Numbers below the panel represent the cell counts spotted on TAP plates. (c) Immunoblot analysis of LIGHT‐HARVESTING COMPLEX STRESS RELATED (LHCSR) proteins in the *det1 dos* mutants. Total cell extracts were prepared from cells exposed to low‐light (L) or high‐light (H) conditions for 4 h. AtpB levels serve as a loading control. (d) Schematic representation of *Bill* insertions in the *CrCO* and *NFYB* genes in the *det1 dos* mutants. (e) Genomic DNA sequences around the *Bill* insertion sites in the *det1 dos* mutants. The characteristic *Bill* sequences, including terminal inverted repeats and target site duplications, are depicted in *red* and *blue*, respectively.

When the F2 *det1* cells were backcrossed with WT, while the majority of the F3 *det1* progeny showed the original *det1* phenotype, characterized by slow growth under LL, some did not (Figs 2a, S3A). Even among the former slow‐growing progeny, some formed significantly larger colonies than the others (Fig. 2a). We encountered similar phenomena with the *phot det1* mutant, leading us to suspect that it was due to the *det1* mutation. Subsequently, we found that the fast‐growing *det1* progeny did not show high NPQ when grown under LL (Fig. S3B), suggesting that their fast‐growing phenotype may have arisen due to additional genetic mutation(s) in the components for NPQ induction. We therefore independently isolated five *det1* mutant clones that exhibited a fast‐growing phenotype and named the possible additional mutant alleles *DET‐ONE SUPPRESSOR 1*–*5* (*dos1*–*dos5*). All of these *det1 dos* mutants exhibited faster growth (Fig. 2b), decreased expression of LHCSRs (Fig. 2c), and lower NPQ (Fig. S3C) under LL conditions compared with the parent *det1* mutant.

### The *dos1–3* and *dos4–5* are linked to *crco* and *nfyb*

To examine the genetic relationships among the *dos* mutations, genetic linkage analyses were conducted. The *det1 dos1* mutant was crossed with the *det1 dos2–dos5* mutants. Crosses between *det1 dos1* and *det1 dos3* failed to produce any viable progeny. None of the progeny from crosses between *det1 dos1* and *det1 dos2*, nor between *det1 dos2* and *det1 dos3*, exhibited high NPQ under the LL conditions, suggesting that *dos1*, *dos2*, and *dos3* are genetically linked. Conversely, a substantial proportion of the progeny from crosses between *det1 dos1* and either *det1 dos4* or *det1 dos5* displayed high NPQ without HL pretreatment, indicating that neither *dos4* nor *dos5* is allelic to *dos1*. Furthermore, none of the progeny from the *det1 dos4* and *det1 dos5* cross showed high NPQ, implying that *dos4* and *dos5* are also genetically linked. These genetic analyses revealed that the mutations can be classified into two linkage groups: one comprising *dos1*, *dos2*, and *dos3* and another comprising *dos4* and *dos5* (Table S2).

Because CrCO/NF‐Ys transcription factor complex has been suggested to positively regulate *LHCSR* transcription (Tokutsu *et al*., 2019a), we first generated *det1 crco*, *det1 nfyb*, and *det1 nfyc* double mutants to examine whether this transcription factor complex is involved in the elevated LHCSR expression observed in the *det1* mutant under LL conditions. The results indicated that all of these double mutants abolished the constitutive accumulation of LHCSR1 and LHCSR3 expression (Fig. S4A), which was accompanied by the rescue of the slow growth phenotype of *det1* (Fig. S4B) with reduced NPQ (Fig. S4C). The induction of LHCSR3 under HL was still observed in the double mutants (Fig. S4A), suggesting that at least part of the HL‐induced LHCSR3 expression is regulated by a pathway distinct from CrCO/NF‐Ys, likely through the carbon concentration mechanism, as recently reported (Ruiz‐Sola *et al*., 2023).

Subsequently, since the phenotype of *det1* was suppressed by *dos* mutations (Fig. 2b), the genetic linkages between the *dos* mutants and the *crco*, *nfyb*, and *nfyc* mutations were examined. In crosses between *det1 dos1* and *det1 nfyc*, progeny displaying high NPQ were identified, indicating that *dos1* and *nfyc* mutations are not allelic. Conversely, none of the progeny exhibited high NPQ from crosses between *det1 dos1* and *det1 crco*, suggesting a genetic linkage between *dos1* and *crco* mutations. Similarly, crosses between *det1 dos5* and *det1 nfyb* yielded no progeny with high NPQ, indicating a genetic linkage between *dos5* and *nfyb*. These results indicated that the *dos1–3* mutations were linked to *crco*, and the *dos4–5* mutations to *nfyb*, with no involvement of other mutations (Table S2). Further PCR analysis revealed that the amplified fragments of the *CrCO* gene in *det1 dos1*, *det1 dos2*, and *det1 dos3* mutants were longer than that of the WT (Fig. S5A). Similarly, the amplified fragments of the *NFYB* gene in *det1 dos4* and *det1 dos5* mutants were longer than that of the WT (Fig. S5B). Taken together, the genetic linkage analysis revealed that *dos1*–*dos3* mutants and *dos4*–*dos5* mutants contain insertional mutations in *CrCO* and *NFYB* genes, respectively.

### The *dos* loci carry a nonautonomous TE, *Bill*

To further investigate the mutations in the *dos* loci, multiple regions of the *CrCO* and *NFYB* genes were amplified and sequenced using gene‐specific primers. The results indicated that there were two identical 558‐bp insertions in opposite directions within the *CrCO* gene in *det1 dos1* (Fig. 2d). Remarkably, the *CrCO* gene in the *det1 dos3* mutant and the *NFYB* gene in the *det1 dos4* and *det1 dos5* mutants also had insertions of the nearly identical 558‐bp sequences (Fig. 2d). We found that those 558‐bp DNA fragments were highly similar to the sequence of a previously reported MITE in *C. reinhardtii*, designated as *Bill* in the literature (Craig *et al*., 2023). As in the reported sequence of *Bill*, all the 558‐bp sequences in *CrCO* and *NFYB* contained 17‐bp terminal inverted repeats at both ends and were flanked by 8‐bp target site duplications on both sides (Kim *et al*., 2006) (Fig. 2e). The amplified product of the *det1 dos2* mutant was, however, somehow heterogeneous, and although the insertion site was identified within the first intron of *CrCO*, the sequence of the insertion could not be determined.

**Fig. 3:**
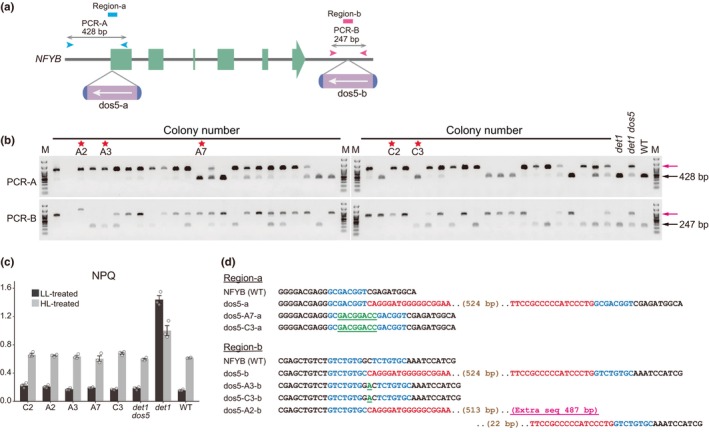
Ongoing *Bill* transposition in the *Chlamydomonas reinhardtii* DE‐ETIOLATED 1 (*det1*) *det1* suppressor5 (*dos5*) mutant. (a) Schematic of *Bill* insertions and target regions for PCR analysis in the *NFYB* gene of the *det1 dos5* mutant. Primer locations for PCR amplification are indicated by *cyan* and *magenta* arrowheads for PCR‐A and PCR‐B, respectively. Expected sizes of PCR products from wild‐type (WT) genomic DNA are shown. *Cyan* and *magenta* bars represent the sequenced regions shown in (d). (b) PCR analysis of the *NFYB* gene in 45 single colonies of the *det1 dos5* mutant after subculturing. Representative clones with (A2, A3, and C2 in PCR‐A; A2, A7, and C2 in PCR‐B; *red‐arrowed position*) and without (A7 and C3 in PCR‐A; A3 and C3 in PCR‐B; *black‐arrowed position*). The clones selected for nonphotochemical quenching (NPQ) (c) and sequence (d) analysis are marked with *red stars*. (c) NPQ analysis of clones that lost *Bill* insertions. NPQ values after exposure to low light (LL) or high light (HL) for 4 h are shown. Data represent mean ± SEM (*n* = 3). (d) Nucleotide sequences of selected clones with and without *Bill* insertions. Nucleotides corresponding to *Bill*'s terminal inverted repeats, target site duplications, extra nucleotides, and the extralong insertion are shown in *red*, *blue*, *green underlined*, and *magenta underlined*, respectively.

When the 5′ and 3′ regions of the *NFYB* gene in the *det1 dos5* mutant, which harbor the *dos5‐a* and *dos5‐b* insertions, respectively (Fig. 3a), were PCR‐amplified using templates from 45 randomly selected mutant colonies, including A2, A3, and C2, long PCR products corresponding to the fragments with the *Bill* insertion were predominantly identified (PCR‐A; Fig. 3b). However, some colonies, such as A7 and C3, displayed short products corresponding to the fragments without the *Bill* insertion (Fig. 3b). Given that these *det1 dos5* mutants underwent single‐colony isolation, it is probable that the cells displaying short PCR products initially carried the insertion but have since lost it. This hypothesis was further supported by similar observations using *det1 dos1*, in which some single colonies exhibited short PCR products without the *Bill* insert in the *CrCO* gene (Fig. S6A,B). Importantly, such clones lacking the *Bill* insert exhibited the same low NPQ phenotype as those retaining the *Bill* insert (Fig. 3c). This was confirmed by sequencing the regions in which *Bill* was excised (Fig. S7), revealing the footprints of extra nucleotides or extralong insertion (Fig. 3d). These results suggest that *Bill* transposition is ongoing in the cultures of *det1 dos* mutants.

### *Bill* transposition is enhanced in the *det1*

**Fig. 4:**
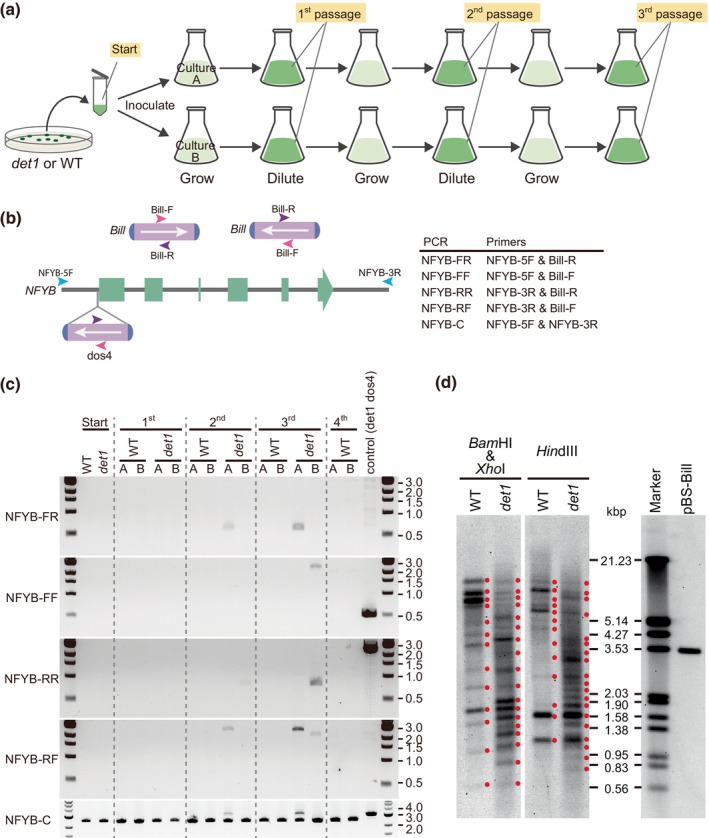
Enhanced *Bill* transposition in the DE‐ETIOLATED 1 (*det1*) mutant in *Chlamydomonas reinhardtii*. (a) Workflow for sampling the *det1* genome from passaged cultures. Cells were suspended in tris‐acetate‐phosphate (TAP) medium at *c*. 2 × 10^6^ cells ml^−1^, referred to as the ‘Start culture’. This culture was inoculated into two flasks (A and B) containing TAP medium at a 1 : 10 000 dilution. Cells were cultured with shaking under 40–50 μmol photons m^−2^ s^−1^ light. Once the culture reached 2–7 × 10^6^ cells ml^−1^, samples were collected for genomic PCR, and the culture was diluted again at 1 : 10 000 to continue growth. This process was repeated to obtain 1^st^, 2^nd^, and 3^rd^ passage samples for genomic PCR, while for wild‐type (WT), samples were collected up to the 4^th^ passage. (b) Schematic representation of the *NFYB* gene showing *Bill* insertions and primer locations for PCR analysis (*left*). A table listing the PCR product names and their corresponding primers (*right*). (c) Genomic PCR analysis of *Bill* insertions in the *NFYB* gene from 1^st^ to 4^th^ passage samples. PCR product names are indicated on the left side of the gels. Amplification of the *NFYB* gene (NFYB‐C) serves as a control to verify that equal amounts of template were loaded. (d) Southern blot analysis of *det1* mutant and WT. Total genomic DNA was digested with *Hin*dIII (*right*) or a combination of *Bam*HI and *Xho*I (*left*) and probed for *Bill*. *Red dots* mark the positions of the bands. A DIG‐labeled marker and a positive control (pBS‐Bill) are shown in the *far‐right panel*.

As mentioned previously, *Bill* was integrated into the *CrCO* and *NFYB* genes in *det1 dos1*–*dos3* and *det1 dos4*–*dos5* mutants, respectively. Once integrated, *Bill* transposed from its original position to other locations. This led us to a hypothesis that the transposition activity of *Bill* is enhanced in the *det1* background. To test this hypothesis, the transposition frequency of *Bill* in the *det1* mutant was compared with that in the WT. Both the *det1* mutant and the WT were inoculated in liquid media in duplicates. When the cell concentration reached 2–7 × 10^6^ cells ml^−1^, portions of the cultures were recovered and diluted into fresh media, repeating this process at least three times (Fig. 4a). Genomic PCR was then performed using primers specific to the *Bill* internal sequence and the *NFYB* flanking sequences (Fig. 4b). In the WT, no PCR fragments were detected in the *NFYB* gene up to the fourth passage (approximately after 51–53 divisions) (Fig. 4c). In *det1*, however, the PCR products were detected as early as the second passage (approximately after 24–27 divisions) in both duplicates (flask A and B), demonstrating that more frequent *Bill* insertions occur in the *NFYB* (Fig. 4c). Similar results were obtained for the *CrCO* gene, in which *Bill* insertions were identified as early as the second passage (Fig. S8B). Finally, Southern blot analysis compared the copy number of *Bill* in the *det1* and the WT genomes using the *Bill* sequence as a probe. Thirteen bands were detected in the WT genome when digested with *Bam*HI and *Xho*I, as well as with *Hin*dIII (Fig. 4d). By contrast, the band pattern in the *det1* mutant was markedly complex, with an increased number of bands, supporting the conclusion that *Bill* transposes more frequently in the *det1* background than in the WT.

## Discussion

Although numerous studies have effectively described the conditions under which TEs are mobilized, including changes in epigenetic markers and modifications in chromatin organization (Slotkin & Martienssen, 2007; Zeh *et al*., 2009), the molecular mechanisms both upstream and downstream of these changes remain largely unclear. The findings in the current study suggest that DET1, an E3 ubiquitin ligase component, plays a role in not only restricting the induction of LHCSR expression but also maintaining the repression of *Bill* transposition under nonstress conditions. This sophisticated mechanism potentially enables the photosynthetic organism to rapidly acclimate to short‐term environmental fluctuations, such as daily weather changes, while simultaneously enhancing its capacity for long‐term adaptation to sustained environmental shifts, such as climate change. The frequent insertions of *Bill* observed in NPQ‐regulating transcription factors in *det1* suppressors (*dos*) underscore this dual role.

The involvement of DET1 in epigenetic modifications has been previously suggested in land plants. Benvenuto *et al*. (2002) reported that DET1 binds to nonacetylated N‐terminal tails of the core histone H2B in *Arabidopsis*. A subsequent study revealed that dark‐grown *det1* mutant plants exhibited condensed heterochromatin in cotyledon cells, leading the researchers to propose that DET1 mediates light‐triggered heterochromatin reorganization, which potentially underlies the transcriptional reprogramming necessary for de‐etiolation (Bourbousse *et al*., 2015). Although *Chlamydomonas* and *Arabidopsis* are phylogenetically distant, the core regulatory mechanism may have arisen before their divergence and remained conserved. Indeed, key regulatory components involved in flowering in land plants and photoprotection in green algae, such as the COP1/SPA1 E3 ligase and the CO/NF‐Ys transcription factor, are shared between these species (Tokutsu *et al*., 2019a). Therefore, it is not surprising that DET1‐dependent transcriptional regulation, along with its associated epigenetic regulations, may also be conserved across species. Alternatively, TE mobilization might not be directly regulated by DET1 itself, but rather by its downstream factor(s). For example, in humans, DET1 targets the proto‐oncogenic transcription factor c‐Jun for ubiquitination and degradation in embryonic kidney cells (Wertz *et al*., 2004). Under specific conditions, such as in hippocampal progenitors differentiated from the induced pluripotent stem cells derived from Alzheimer's disease patients, TE mobilization was observed upon upregulation of c‐Jun (Scopa *et al*., 2023). This raises another possibility that, in *Chlamydomonas*, TE mobilization could similarly be driven by the upregulation of CrCO/NF‐Ys. This possibility warrants further investigation.

**Fig. 5:**
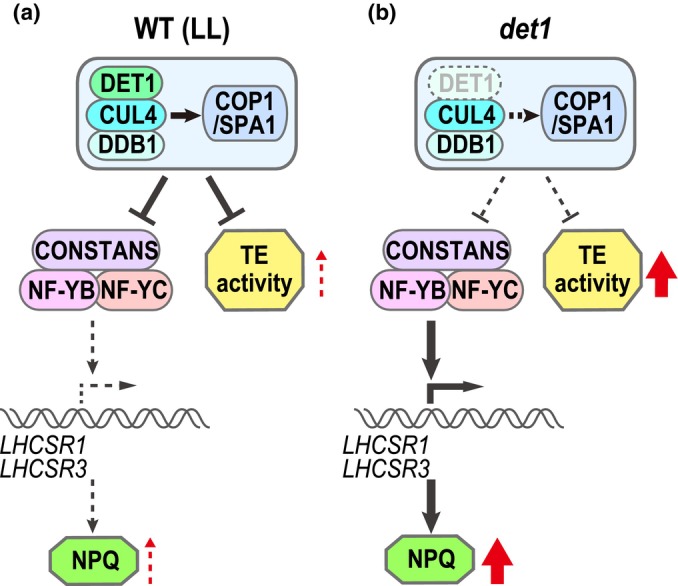
Schematic model for nonphotochemical quenching (NPQ) induction and transposable element (TE) activation via DE‐ETIOLATED 1 (DET1) in *Chlamydomonas reinhardtii*. (a) In wild‐type (WT) under low‐light (LL) conditions, CUL4‐DDB1^DET1^ and COP1/SPA1 E3 ligases suppress both the CrCO/NF‐Ys transcription factor complex and the transposition of *Bill*. (b) In the *det1* mutant, the CUL4‐DDB1^DET1^ and COP1/SPA1 E3 ligases are inactive, resulting in the induction of *LHCSR1/3* expression and the activation of *Bill* transposition. *Arrows* indicate positive regulations, while *blunt‐ended arrows* indicate negative regulations. *Dashed arrow*s indicate interactions that are abolished. *Red upward arrows* signify an increase in the specified activity.

We propose the following sequence of events based on the observations in this study. Under LL conditions in *C. reinhardtii* WT cells, the CUL4‐DDB1^DET1^ and COP1/SPA1 E3 ubiquitin ligases negatively regulate the CrCO/NF‐Ys transcription factor complex, thereby suppressing both the expression of *LHCSR* genes and the mobilization of *Bill* (Fig. 5a). In the absence of DET1 (i.e. in the *det1* mutant), these E3 ligases become inactive, allowing CrCO/NF‐Ys to remain active and to promote *LHCSR* gene expression. Concurrently, *Bill* is allowed to mobilize within the genome, potentially enhancing genomic plasticity (Fig. 5b). A previous study have shown that *LHCSR* expression is triggered by HL stress, specifically via blue light through the phototropin pathway (Petroutsos *et al*., 2016) and via UV light through the UVR8 pathway (Allorent *et al*., 2016). Since DET1 is involved in transducing HL stress signals that lead to *LHCSR* induction (Aihara *et al*., 2019), it may similarly regulate HL‐induced TE mobilization. Thus, as observed in the absence of DET1, HL stress likely promotes *Bill* mobilization in parallel with NPQ induction. A conceptual model illustrating this coordinated regulation of stress response and genome plasticity is presented in Fig. S9.

Future studies will have to determine whether DET1 specifically suppresses the activity of *Bill* or broadly regulates multiple TEs. Furthermore, deciphering the precise molecular mechanisms underlying TE regulation remains a critical objective – particularly how DET1 suppresses TE mobilization and how such suppression is alleviated in response to stress signals. These processes are likely mediated by shifts in epigenetic markers, as numerous studies have documented stress‐induced genomic modifications via epigenetic pathways (Slotkin & Martienssen, 2007; Zeh *et al*., 2009). Ultimately, further studies will be needed to translate the insights obtained from this green algal model into a more comprehensive understanding of the stress responses across diverse organisms. In this context, examining whether *det1* mutant plants display elevated TE mobilization would be especially informative.

## Competing interests

None declared.

## Author contributions

KF‐K conducted the research and prepared the figures. Both KF‐K and JM conceived the research project, interpreted the results, as well as wrote and revised the manuscript.

## Disclaimer

The New Phytologist Foundation remains neutral with regard to jurisdictional claims in maps and in any institutional affiliations.

## Supporting information


**Fig. S1** Signal transduction pathways involved in nonphotochemical quenching induction in *Chlamydomonas reinhardtii*.
**Fig. S2** Construction of DE‐ETIOLATED1 mutant in the wild‐type background.
**Fig. S3** Nonphotochemical quenching of F3 clones of the *det1* mutant.
**Fig. S4** Mutations in *CrCO*, *NF‐YB*, or *NF‐YC* genes counteract LIGHT‐HARVESTING COMPLEX STRESS RELATED1 protein accumulation in the *det1* mutant.
**Fig. S5** Genomic PCR analysis of the *dos* mutants.
**Fig. S6** Insertions and excisions of *Bill* in the *det1 dos1* mutant.
**Fig. S7** PCR analysis of the *NFYB* gene in selected single‐colony clones of the *det1 dos5* mutant.
**Fig. S8** *Bill* insertions in the *CrCO* gene in the *det1* mutant.
**Fig. S9** Conceptual model for the coordinated regulation of stress responses (nonphotochemical quenching) and genome plasticity (transposable element activation) in *Chlamydomonas reinhardtii*.
**Table S1** Primers used in this study.
**Table S2** Ratio of high nonphotochemical quenching progeny resulting from genetic crosses among *det1* and related mutants (*dos1*–*dos5*, *crco*, *nfyb*, and *nfyc*).
Please note: Wiley is not responsible for the content or functionality of any Supporting Information supplied by the authors. Any queries (other than missing material) should be directed to the *New Phytologist* Central Office.

## Data Availability

All the data supporting the findings of this study are included within the paper.
