# Supplementary material for: From photoprotection to plasticity: transposon activation in the Chlamydomonas det1 mutant
Source: New Phytol. 2025 Aug 7;248(2):807–16. doi: 10.1111/nph.70436 (PMC12445801; doi:10.1111/nph.70436)
Supplement: Supplementary file 1 — Fig. S1 Signal transduction pathways involved in nonphotochemical quenching induction in Chlamydomonas reinhardtii. Fig. S2 Construction of DE‐ETIOLATED1 mutant in the wild‐type background. Fig. S3 Nonphotochemical quenching of F3 clones of the det1 mutant. Fig. S4 Mutations in CrCO, NF‐YB, or NF‐YC genes counteract LIGHT‐HARVESTING COMPLEX STRESS RELATED1 protein accumulation in the det1 mutant. Fig. S5 Genomic PCR analysis of the dos mutants. Fig. S6 Insertions and excisions of Bill in the det1 dos1 mutant. Fig. S7 PCR analysis of the NFYB gene in selected single‐colony clones of the det1 dos5 mutant. Fig. S8 Bill insertions in the CrCO gene in the det1 mutant. Fig. S9 Conceptual model for the coordinated regulation of stress responses (nonphotochemical quenching) and genome plasticity (transposable element activation) in Chlamydomonas reinhardtii. Table S1 Primers used in this study. Table S2 Ratio of high nonphotochemical quenching progeny resulting from genetic crosses among det1 and related mutants (dos1–dos5, crco, nfyb, and nfyc). Please note: Wiley is not responsible for the content or functionality of any Supporting Information supplied by the authors. Any queries (other than missing material) should be directed to the New Phytologist Central Office. [file NPH-248-807-s001.pdf]

***New Phytologist* Supporting Information**

Article title: From Photoprotection to Plasticity: Transposon Activation in the *Chlamydomonas det1* Mutant
Authors: Konomi Fujimura-Kamada and Jun Minagawa
Article acceptance date: 15 July 2025

The following Supporting Information is available for this article:

Fig. S1 Signal transduction pathways involved in nonphotochemical quenching induction in *Chlamydomonas reinhardtii*.

Fig. S2 Construction of DE-ETIOLATED1 mutant in the wild-type background.

Fig. S3 Nonphotochemical quenching of F3 clones of the *det1* mutant.

Fig. S4 Mutations in *CrCO*, *NF-YB*, or *NF-YC* genes counteract LIGHT-HARVESTING COMPLEX STRESS RELATED1 protein accumulation in the *det1* mutant.

Fig. S5 Genomic PCR analysis of the *dos* mutants.

Fig. S6 Insertions and excisions of *Bill* in the *det1 dos1* mutant.

Fig. S7 PCR analysis of the *NFYB* gene in selected single-colony clones of the *det1 dos5* mutant.

Fig. S8 *Bill* insertions in the *CrCO* gene in the *det1* mutant.

Fig. S9 Conceptual model for the coordinated regulation of stress responses (nonphotochemical quenching) and genome plasticity (transposable element activation) in *Chlamydomonas reinhardtii*.

Table S1 Primers used in this study.

Table S2 The ratio of high nonphotochemical quenching progeny resulting from genetic crosses among *det1* and related mutants (*dos1–dos5*, *crco*, *nfyb*, and *nfyc*).

Fig. S1 Signal transduction pathways involved in nonphotochemical quenching induction in *C. reinhardtii*. A. Under LL conditions in wild-type (WT), CUL4–DDB1^{DET1} and COP1/SPA1 E3 ubiquitin ligases suppress the CONSTANS (CrCO)/NF-Ys transcription factor complex, leading to downregulation of *LHCSR1/3* gene expression. **B.** Upon exposure to high light (HL) , including blue and UV light, in WT, the light signal inhibits these E3 ligases, thereby relieving the negative regulation of the CONSTANS (CrCO)/NF-Ys transcription factor complex, leading to elevated nonphotochemical quenching (NPQ). **C.** In the *det1* mutant, CrCO/NF-Ys transcription factor complex remains constitutively active regardless of light signal input due to the inactivation of CUL4–DDB1^{DET1}. This constitutive activation promotes *LHCSR1/3* expression, resulting in high NPQ. *Dashed arrows* indicate regulatory pathways that are disrupted. *Arrows* represent positive regulation, while *blunt-ended arrows* represent negative regulation. *Red upward arrows* indicate elevated activity. *Red asterisks* in **B** and **C** denote regulatory checkpoints, perturbations of which gave rise to outcomes distinct from those observed in **A**.

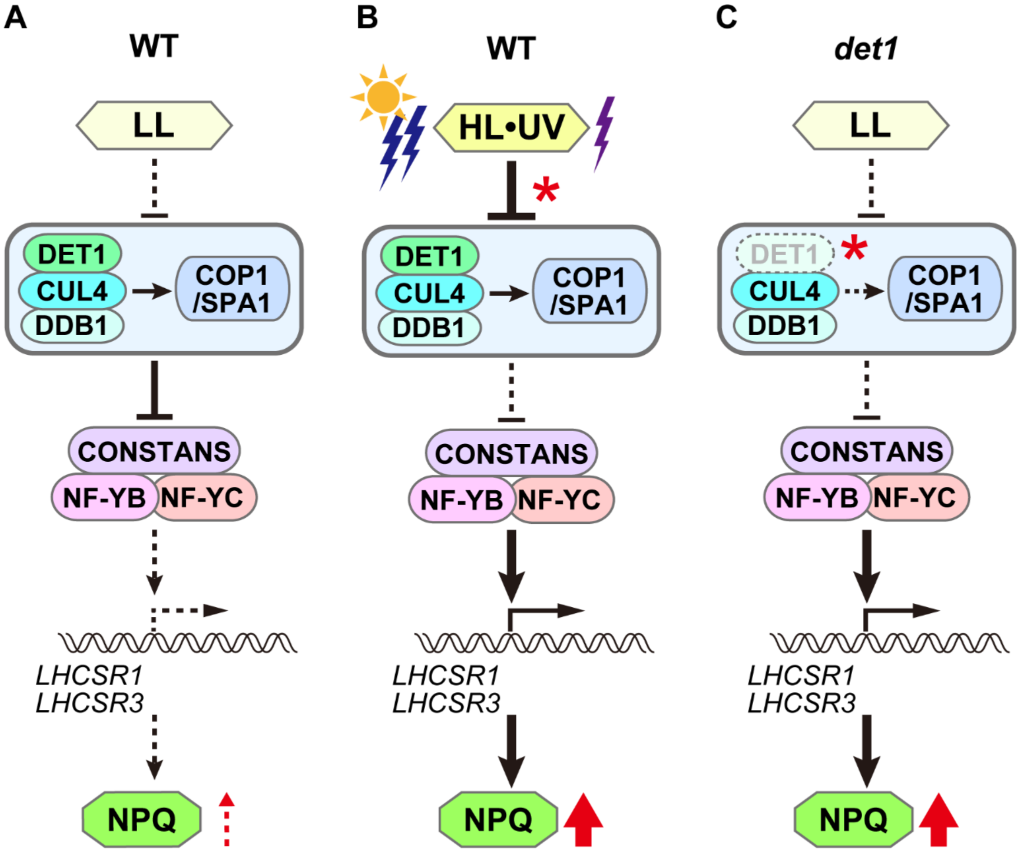

Fig. S2 Construction of DE-ETIOLATED1 mutant in the wild-type background. **A**. Schematic representation of the DE-ETIOLATED1 (*DET1*) mutant created using the CRISPR-Cas9 system. **B**. The *det1* single mutant was generated by crossing *phot det1* double mutant with the wild-type (WT).

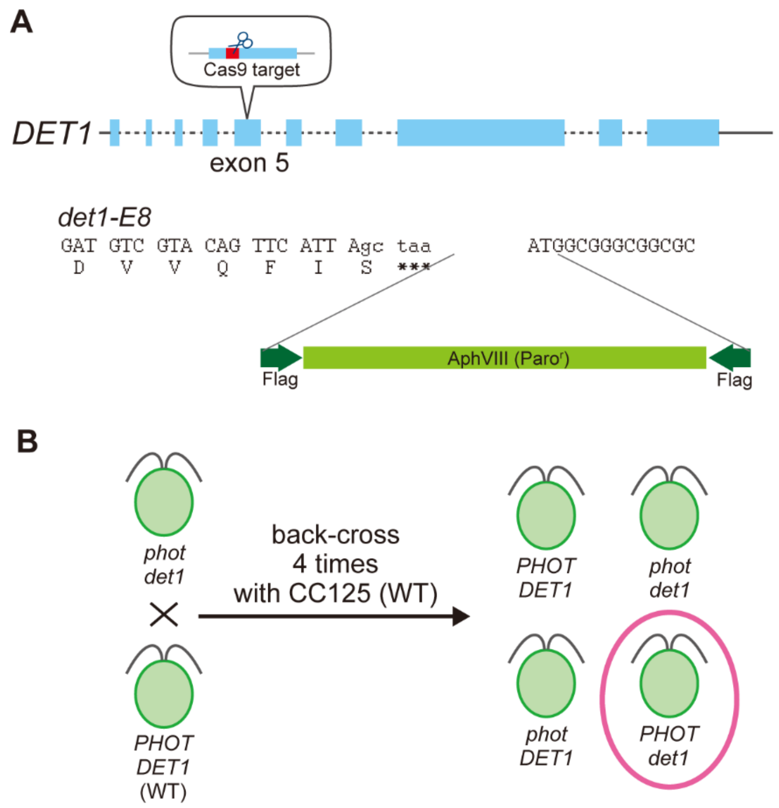

Fig. S3 Nonphotochemical quenching of F3 clones of the *det1* mutant. A. Diagram showing the acquisition process of F3 clones of the *det1* mutant. The genotype of each strain is indicated in parentheses. **B.** Nonphotochemical quenching (NPQ) of the F3 clones. NPQ was measured after 4 h of low light (LL) or high light (HL) illumination. Data are presented as the means ± SEM (n = 3). Relatively well-growing *det1* clones (C3, A3) are marked with *cyan circles*, while poorly growing *det1* clones (D5, D10) are marked with *red circles*. **C.** NPQ of the *det1 dos* mutants. NPQ was measured after 4 h of LL (*black*) or HL (*gray*) illumination. Data are presented as the means ± SEM (n = 3).

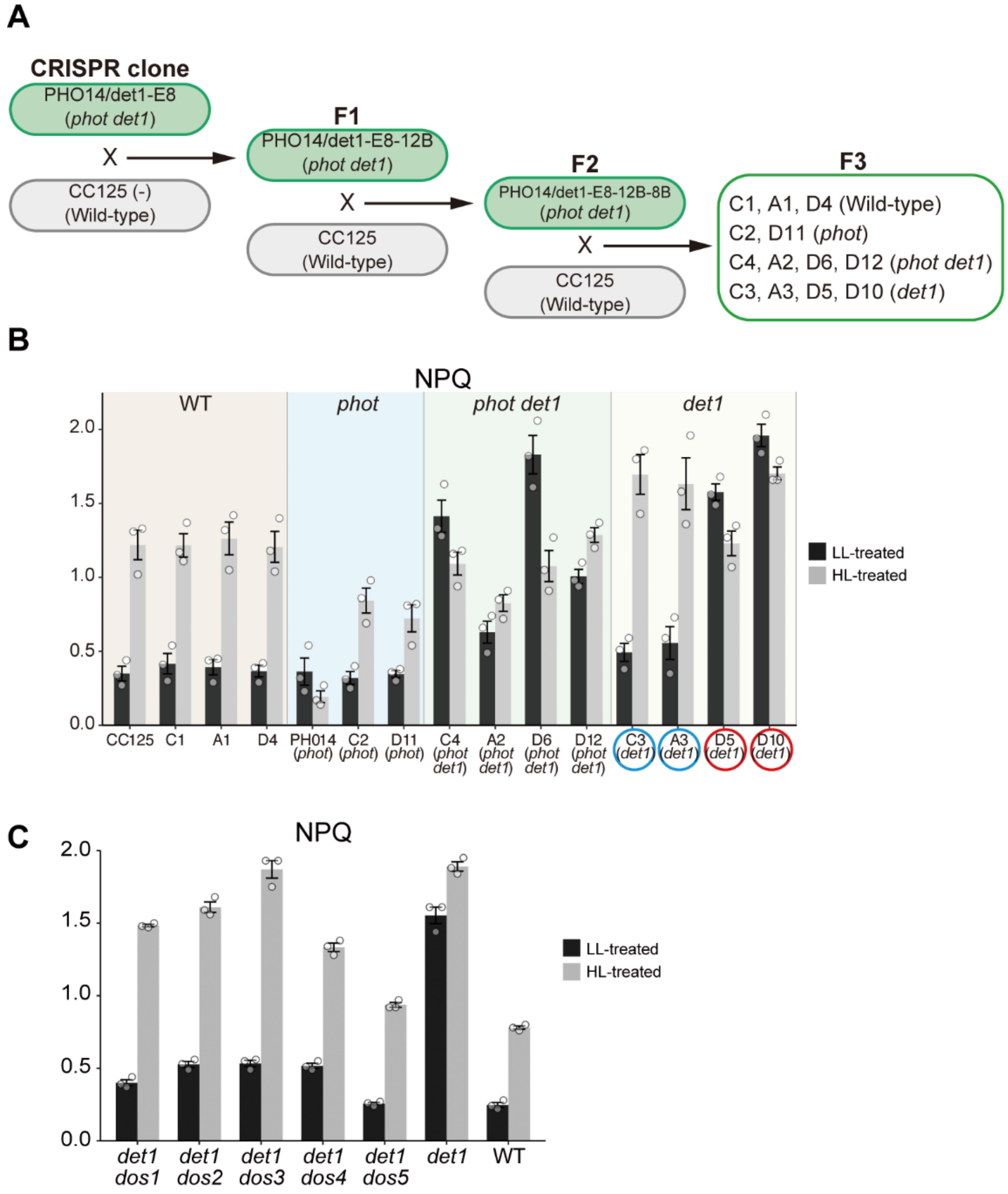

Fig. S4 Mutations in *CrCO*, *NF-YB*, or *NF-YC* genes counteract LHCSR protein accumulation in the *det1* mutant. A. Immunoblot analysis of LHCSR proteins in *det1 crco*, *det1 nfyb*, and *det1 nfyc* double mutants. Total cell extracts were prepared from cells after 4 h of LL or HL illumination. AtpB levels are shown as a loading control. **B.** Growth comparison of *det1 crco*, *det1 nfyb*, and *det1 nfyc* double mutants with the *det1* single mutant and the wild-type (WT). The number of cells indicated below the panel was spotted on a tris-acetate phosphate (TAP) plate and incubated for 4 d under a light intensity of 50 μmol photons m^{-2} sec^{-1}. **C.** NPQ of *det1 crco*, *det1 nfyb*, and *det1 nfyc* double mutants. NPQ was measured after 4 h of LL (*black*) or HL (*gray*) illumination. Data are presented as the means ± SEM (n = 3).

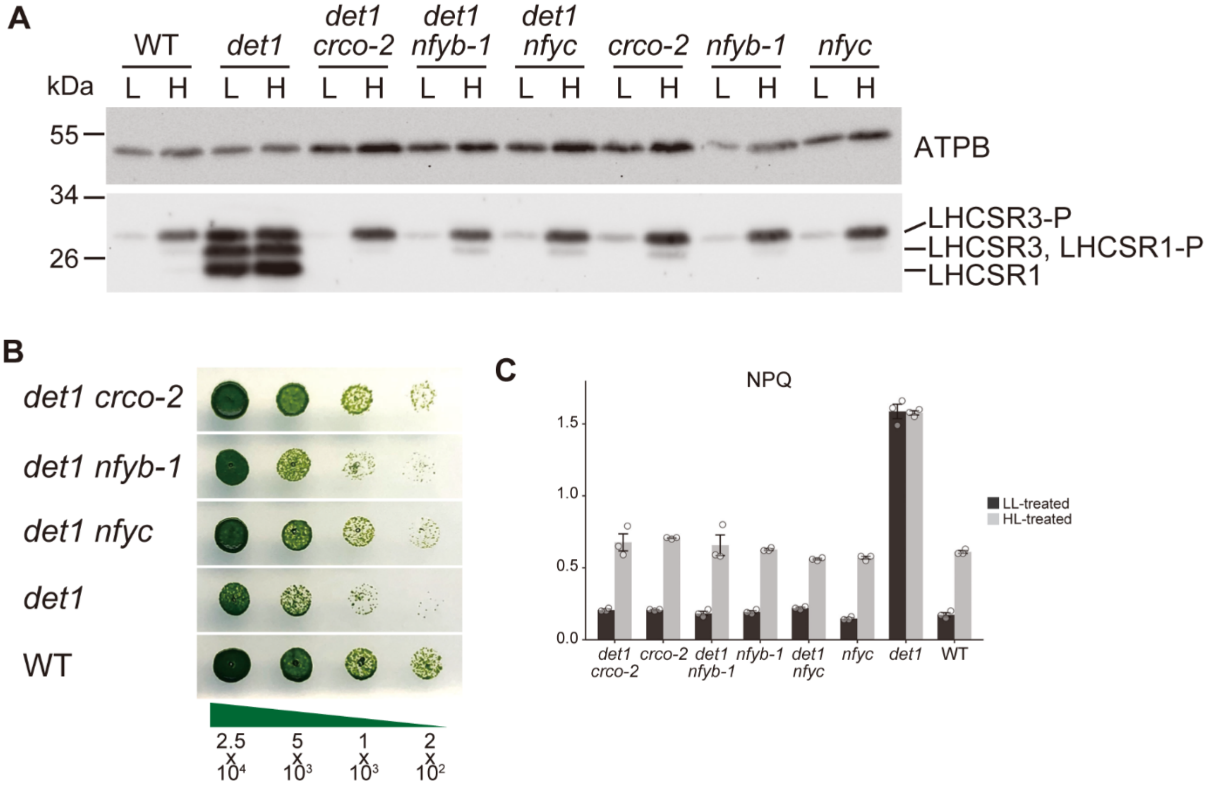

Fig. S5 Genomic PCR analysis of the *dos* mutants. A. Genomic PCR analysis of the *CrCO* gene in *det1 dos1*, *det1 dos2* and *det1 dos3* mutants (*upper panel*). A schematic representation indicates the locations of primers and the PCR products (*lower panel*). The expected sizes of the PCR products for the wild-type (WT) genome are shown. **B.** Genomic PCR analysis of the *NFYB* gene in *det1 dos4* and *det1 dos5* mutants. All other conditions are the same as in **A**.

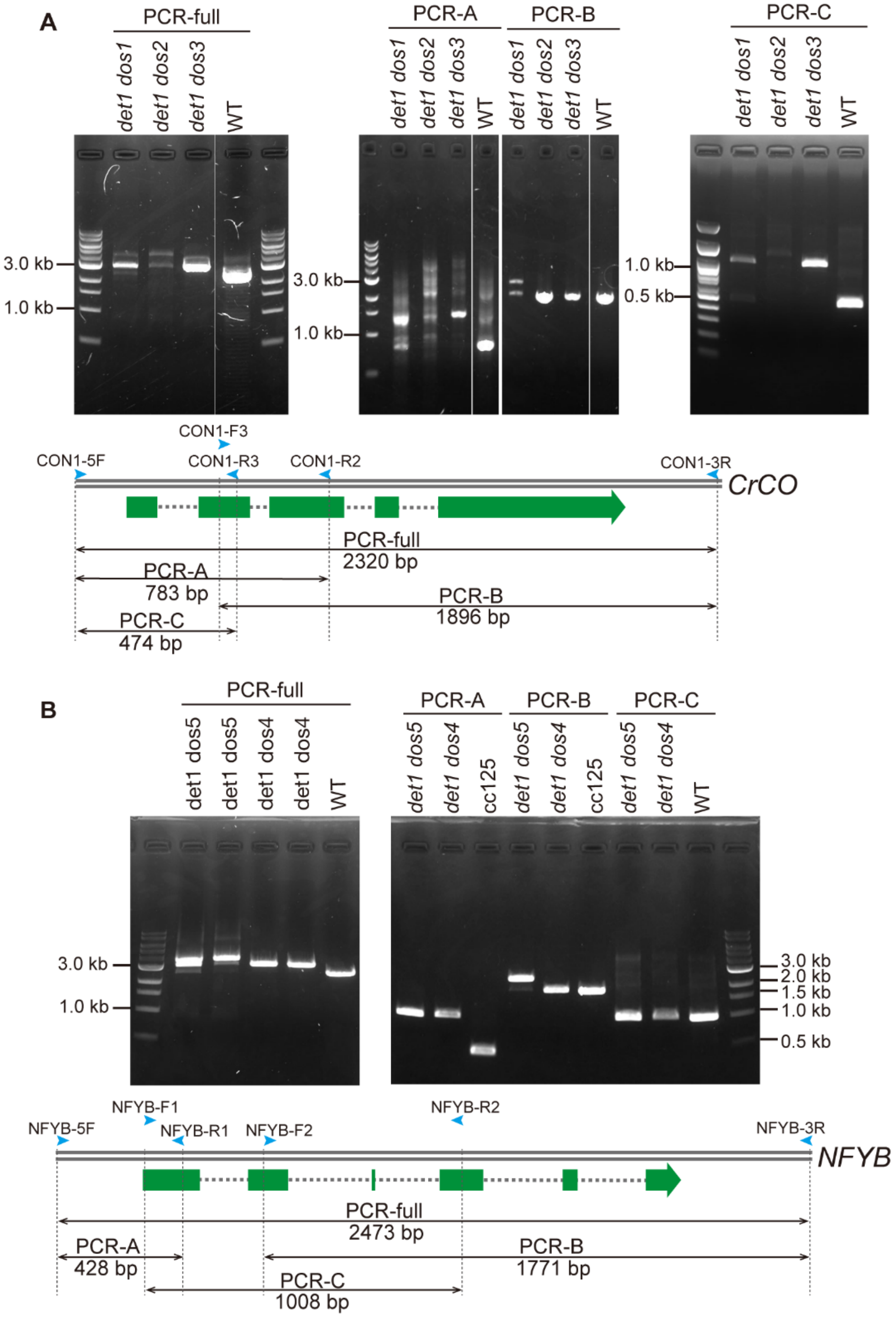

Fig. S6 Insertions and excisions of *Bill* in the *det1 dos1* mutant. A. Locations of *Bill* insertions and target regions for PCR analysis in the *CrCO* gene of the *det1 dos1* mutant. Primer locations used for PCR amplification are indicated by *cyan* and *magenta arrow heads* for PCR-C and PCR-D, respectively. The expected sizes of PCR products for wild-type (WT) genomic DNA are shown. **B.** PCR analysis of the *CrCO* gene in 14 single-colonies of the *det1 dos1* mutant after repeated subculturing, using the primer sets shown in **A**.

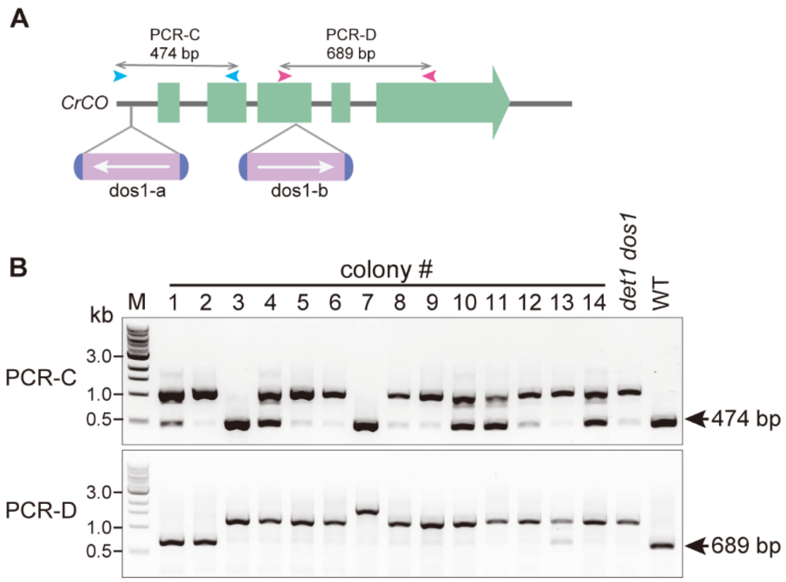

Fig. S7 PCR analysis of the *NFYB* gene in selected single-colony clones of the *det1 dos5* mutant. The amplified DNA bands circled in *yellow* were extracted from the gel and subjected to sequence analysis (see Fig. 3c). A table summarizing the presence (+) or absence (-) of *Bill* insertions is shown (*below*).

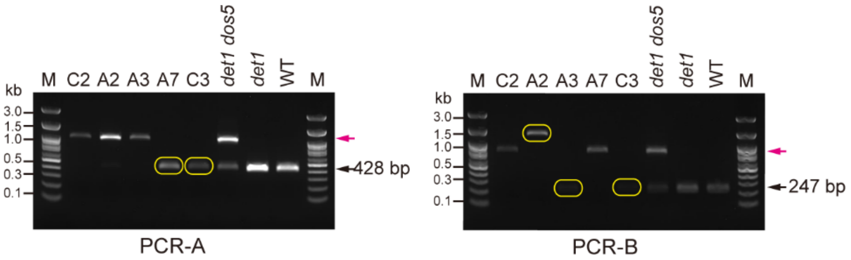

Clone #	A2	A3	A7	C2	C3
Bill insertion of dos5-a	+	+	-	+	-
Bill insertion of dos5-b	++?	-	+	+	-

Fig. S8 *Bill* insertions in the *CrCO* gene in the *det1* mutant. A. Schematic representation of the *CrCO* gene showing *Bill* insertions and primer locations used for PCR analysis (*left*). A table listing the PCR product names and their corresponding primers (*right*). **B.** Genomic PCR analysis of *Bill* insertions in the *CrCO* gene from 1st to 3rd passage samples. The names of PCR products are indicated on the left side of the gels. Amplification of the *CrCO* gene (CON-C) serves as a control to verify that equal amounts of template were loaded.

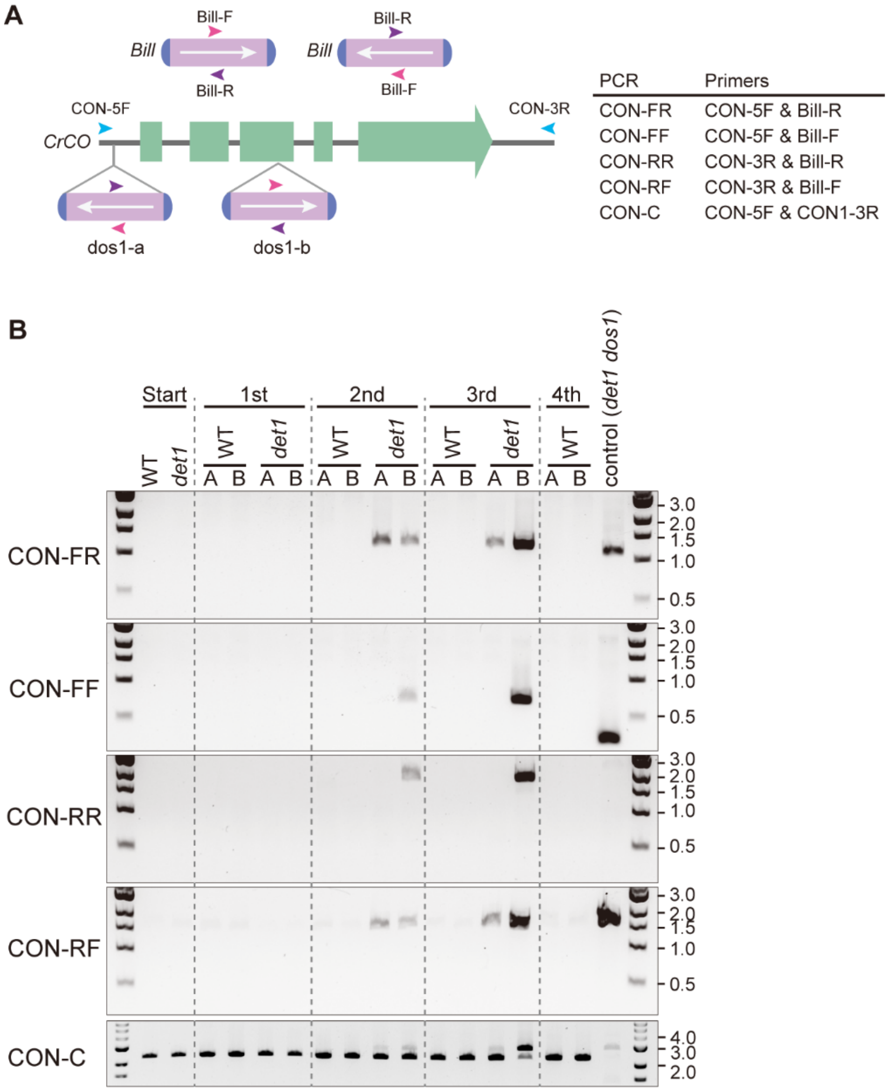

Fig. S9 Conceptual model for the coordinated regulation of stress responses (nonphotochemical quenching) and genome plasticity (transposable element activation) in *C. reinhardtii*. A. Under low light (LL) non-stress conditions in the wild-type (WT), CUL4–DDB1^{DET1} and COP1/SPA1 E3 ubiquitin ligases suppress both *LHCSR1/3* gene expression and *Bill* transposition. **B**. Under LL conditions in *det1* mutant, inactivation of the CUL4–DDB1^{DET1} leads to derepression of the CrCO/NF-Ys transcription factor, resulting in upregulation of *LHCSR1/3* expression and activation of *Bill* transposition. **C**. Under high light (HL) stress conditions in WT, blue or UV light inactivates these E3 ligases, leading to enhanced *LHCSR1/3* expression (nonphotochemical quenching (NPQ) response) and *Bill* transposition (genome plasticity). *Dashed arrows* indicate regulatory pathways that are disrupted. *Arrows* represent positive regulation, while *blunt-ended arrows* represent negative regulation. *Red upward arrows* indicate elevated activity. *Red asterisks* in **B** and **C** denote regulatory checkpoints, perturbations of which gave rise to outcomes distinct from those observed in **A**.

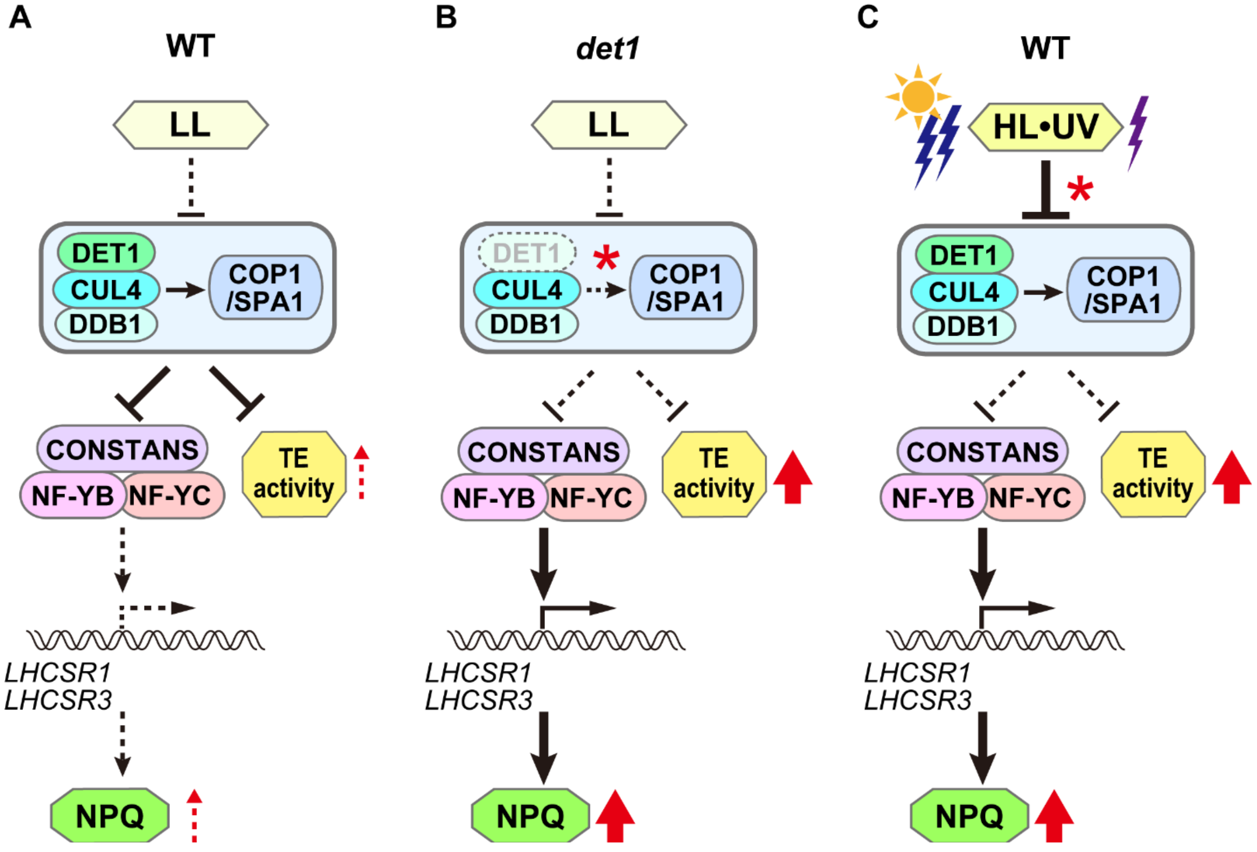

Table S1 Primers used in this study

Name	Sequence	Application
tag-V2-F	TTAGCTAAGCCTCCCCAAAGCCTGGCCAGGGTCTAG	Donor of CRISPR-Cas9
tag-V2-R	CTAGACCCTGGCCAGGCTTTGGGGAGGCTTAGCTAA	Donor of CRISPR-Cas9
DET1-check-F	ACTCGTCGATGGAACTGGTC	PCR screening for *det1* mutants created by CRISPR-Cas9
DET1-check-R	CAGCACGCAGATGAGGTCCT	PCR screening for *det1* mutants created by CRISPR-Cas9
FLAG ver2-check-F	CCCAAAGCCTGGCCAGGGTC	PCR screening for *det1* mutants created by CRISPR-Cas9
FLAG ver2-check-R	CCAGGCTTTGGGGAGGCTTAG	PCR screening for *det1* mutants created by CRISPR-Cas9
CON1-5F	CATGGGGTGCTTGCTGGAC	PCR in Fig. S5A (PCR-full, PCR-A, and PCR-C), Fig. S6B (PCR-C), and Fig. S8A
CON1-F2	CATGGGGTGCTTGCTGGAC	PCR for pBSIIKS-Bill plasmid construction
CON1-F3	GCTGCGCTCTACTGCAAGTG	PCR for Fig. S5A (PCR-B)
CON1-F4	CGTGGTGCCCGTCATGTC	PCR for Fig. S6B (PCR-D)
CON1-3R	GCGCACGGGACCAGCTATAAC	PCR for Fig. S5A (PCR-full and PCR-B) and Fig. S8A
CON1-R2	CGGACATGACGGGCACCAC	PCR for Fig. S5A (PCR-A)
CON1-R3	TGGCAAGCCTCGCACATGTG	PCR for Fig. S5A (PCR-C) and Fig. S6B (PCR-C)
CON1-R6	GAAGAACAGCGCTGACGCCTG	PCR for Fig. S6B (PCR-D)
NFYB-5F	ACGCGGTAATCGTGCTTACAG	PCR for Fig 4b, Fig. S5B (PCR-full, PCR-A), and Fig. S6A (PCR-A)
NFYB-3R	TCGCGATGGATTTGCACAGAG	PCR for Fig 4b and Fig. S5B (PCR-full and PCR-B)
NFYB-F1	GACGGTCGAGATGGCAACAG	PCR for Fig. S5B (PCR-C)
NFYB-F2	TTGGGCTTTGAGGAGTACCTG	PCR for Fig. S5B (PCR-B)
NFYB-F3	ACTTGCGCAAGGCAACTTACTG	PCR for Fig. S6A (PCR-B)
NFYB-R1	ACCGTCTCCTTTGCATCCTTG	PCR for Fig. S5B (PCR-A) and Fig. S6A (PCR-A)
NFYB-R2	CTTTGCCTCCGCGTTGGCAC	PCR for Fig. S5B (PCR-C)
NFYB-R3	TAGGGTGCGGTGCGGTAGAG	PCR for Fig. S6A (PCR-B)
Bill-F	CACAACCAGCGGTGTTGTCGC	PCR for Fig. S8A and Fig. S8B
Bill-R	GCGACAACACCGCTGGTTGTG	PCR for Fig. S8A and Fig. S8B
BamHI-Bill-Fw	TTTTGGATCCAGGGATGGGGGCGGAAG	PCR for pBSIIKS-Bill construction and template for DIG-labeled probe generation
EcoRI-Bill-Rv	TTTTGAATTCAGGGATGGGGGCGGAATG	PCR for pBSIIKS-Bill construction and template for DIG-labeled probe generation

Table S2 Ration of high nonphotochemical quenching progeny resulting from genetic crosses among *det1* and related mutants (*dos1–dos5*, *crco*, *nfyb*, and *nfyc*) Click here to enter text.

cross	Number of high nonphotochemical quenching (NPQ) progeny / number of tested progeny
det1 dos1 x *det1 dos2*	0 / 20
det1 dos1 x *det1 dos3*	ND*
det1 dos1 x *det1 dos4*	17 / 61
det1 dos1 x *det1 dos5*	13 / 40
det1 dos2 x *det1 dos3*	0 / 20
det1 dos4 x *det1 dos5*	0 / 40
det1 dos1 x *det1 nfyc*	5 / 20
det1 dos1 x *det1 crco*	0 / 20
det1 dos5 x *det1 nfyb*	0 / 56

*ND: not determined
